# Progress in Surgery

**Published:** 1899-07-15

**Authors:** 


### PROGRESS IN SURGERY.

### HERNIA.

**The Radical Cure of Femoral Hernia.**—Dr. Silver[1] has recently written on this subject. He reviews the various methods which have been adopted, classifying them into four groups. These are: (1) Methods which deal with the sac alone; (2) those which have in view the closure or obliteration of the internal ring and canal by relaxing Poupart's ligament, *e.g.*, those of Fabricius, Fowler, and Delagenière; (3) those which have in view the restoration of the canal to its normal relations, *e.g.*, that of Bassini; (4) those which make an attempt to close the rings or canal with some substance which will plug the canal, or act as a barrier at the internal or external ring—Sulzer, Cheyne, Moullin, &c. The author describes his own method, which he alludes to as the inguinal method. The incision is a four-inch one, just above and parallel to Poupart's ligament. This incision is strongly recommended for radical cure, and also for cases of small strangulated femoral hernia. The external oblique aponeurosis is then incised along the same line, and the neck of the femoral sac exposed from above. The neck and the rest of the sac are loosened and drawn out of the canal, opened, and the contents dealt with, and the opening closed again. The fundus is then grasped with a clamp and gently rolled like a scroll in a longitudinal direction in order to avoid traction on the bladder, until it is pressed against the peritoneum. A suture through the folds prevents unrolling, and then the rolled sac is sutured to Poupart's ligament above and to Cooper's ligament below. This brings the mass down over the internal femoral ring. The close connection of the bladder has to be borne in mind, as it has been injured in operation for femoral hernia. In separating the sac its covering of fascia and vessels must not be stripped off, and the rolling done loosely, lest its nutrition be impaired.

McArdle[2] performs and advises the following operation for the radical cure under discussion. The incision is semilunar, beginning above the middle of Poupart's ligament, curved downwards, and ending a finger's breadth above the spine of the pubes. The flap is reflected up and the femoral ring and canal thus fully exposed and plenty of room given for manipulations. The sac is cut across an inch and a half below Poupart's ligament, and having been emptied is seized in a long-bladed clip forceps passed through the internal oblique from without and along the femoral canal in front, and drawn through the small opening in the oblique muscle, and fixed there by a suture. Next a suture with a curved needle at each end is passed deeply through the pectineus muscle transversely, leaving a loop on its deep surface. The needles are now passed through the femoral canal and made to pierce the internal oblique. Next the pectineus muscle is reflected up as a curved flap and drawn well up by the above suture, which is tied. The skin wound is then closed.

We think it would be an improvement to displace the sac by the inversion method, *i.e.*, by passing the forceps into the peritoneal cavity and out through the sac. The extremity of the latter is then seized and withdrawn as above. This is Kocher's most recent method of displacement of the sac internal to the ring, as in inguinal hernia. Bull and Coley[3] report results nearly perfect from high ligation of the sac and closure of the femoral canal by a purse-string suture (Cushing's). A curved needle with kangaroo tendon suture is passed through Poupart's ligament, then through fascia and pectineus muscle, brought out, and several more folds taken of the same tissue forming the floor of the canal, then through the fascia over the femoral vein and out again through Poupart's ligament. In other cases they found Bassini's method equally successful. Femoral hernia is rare before puberty, but these surgeons have operated on 20 such cases, and as femoral hernia seems to be incurable by trusses they recommend the operation in all cases if the patients are suitable surgical subjects.

**Cases of Special Interest.**—De Santi[4] records three of these. In the first a female, æt. 21, had symptoms of strangulated hernia, and examination showed the presence of both an inguinal and a femoral hernia. The history, however, showed that it was the inguinal one which had become strangulated, for that one had become painful and irreducible. The strangulated gut was reduced, and some days later both herniæ radically cured through a single vertical incision. The femoral hernia contained the vermiform appendix and some omentum. The second case was one of reducible left inguinal hernia in a man of 24, which was subjected to the operation of radical cure. The corresponding half of the scrotum was imperfectly developed, and the testicle and epididymus were absent; but the spermatic cord was well formed and traceable to the bottom of the hernial sac, and the vas was patent up to within an inch of its termination. It is suggested that there had been a testicle which had degenerated into a very small mass of fibrous tissue. The third case was one of strangulated interstitial hernia, and there was a myxomatous condition of the testis and cord. The additional diverticulum of the hernial sac extended between the internal and external oblique muscles, which is the commonest form. Da Costa[5] has also reported three hernia cases with unusual complications. The first was remarkable from being of enormous size. It was operated upon and reduced with great difficulty, and a sort of Bassini's operation done. A year and a half after there was slight recurrence, but a truss effectually retained it. The second case was remarkable, in that a purulent clot containing staphylococci was removed in one of the veins of the cord. The hernia was strangled, and taxis had been used. Da Costa asks whether the phlebitis was secondary to gonorrhœa, which the man was subsequently found to have, or to taxis. The third case was a strangulated inguinal hernia in a patient with a cough, who had had a cauterisation of the rectum performed for prolapsus ani a few weeks previously. Manipulation of the hernia yielded a crackling sensation. At the operation this was found to be due to air in the mesocolon, large bowel occupying the sac. Similar emphysema was found in all the mesocolon and mesorectum. The air appeared to have passed in through an ulcerated area of the rectum produced by the cautery. From this ulcer air could be expelled by pressure on the bowel. A tube was passed through this opening into the subserous tissue and gradually the emphysema subsided, and recovery followed. Stanmore Bishop[6] relates a case in which he stripped the mesentery from a portion of bowel when separating adhesions to the sac. As the bowel retained its colour it was returned into the abdomen, and no untoward result followed, showing that the anastomoses of vessels on the bowel wall may serve to maintain nutrition.

Delagenière[6] records a case in which on each side a herniated testicle was found in the inguinal region of a supposed female. The patient was a big, thin person of 27. There was a vagina two inches long, and normal vulvar structures except for deficient hair. No uterus or ovaries were made out. The skeleton and muscular structures were of masculine type, the breasts like those of a girl just before puberty. No beard. The tastes and occupations were feminine.

**Interstitial Hernia.**—McAdam Eccles,[7] discussing the causation of this form of hernia, points out that its occurrence is relatively more frequent in the female than in the male. He says that in 1,000 inguinal herniæ in females there will be six of the interstitial variety, whereas in 1,000 such in the male only one will be interstitial. It is thus clear that mere failure of descent of the testicle is not the sole cause of interstitial hernia, and it has not been demonstrated to be associated with congenital herniation of the ovaries. What is the common factor in both sexes? It appears that in nearly every case there is either a patent processus vaginalis or canal of Nuck. The patent processus allows the descent of the viscera, and the abnormal situation of the testis prevents the descent into the scrotum. The viscera therefore travel in the direction of least resistance. In the female the external abdominal ring is smaller than in the male, and probably blocks the way as the testis does in the male. Two other causes are mentioned, viz., an ill-fitting truss which does not press upon the internal abdominal ring, but only upon the external ring; and *reduction en masse*. The dislocated sac forms in the latter case what may be called a traumatic interstitial hernia. When the sac of an interstitial hernia lies between the internal and external oblique muscles, a flattened oval swelling is formed, above and parallel to Poupart's ligament usually, but sometimes vertical. If reduced and then brought down again by successive coughs, the viscera descends and appears to gradually dissect up the plane of the abdominal wall. If there is a scrotal part a groove will separate that portion from the other. When the sac is superficial to the external oblique the swelling will be much the same as in the last variety, but it can be traced to an enlarged external ring, and no hardening of muscle over it can be produced. It has to be distinguished from femoral hernia.

[1] Vide Med. Chron., Dec., 1898. [2] Dublin Jour. of Med. Sci., Dec. 1898. [3] Annals of Surgery, Nov., 1898. [4] Clin Jour., Dec., 1898. [5] Annals of Surgery, Feb., 1899. [6] Brit. Med. Jour., Mar., 1899. [7] Lancet, Dec., 1898.

